# Pediatric hypertension: an updated review

**DOI:** 10.1186/s40885-020-00156-w

**Published:** 2020-12-01

**Authors:** Mohd Ashraf, Mohd Irshad, Nazir Ahmed Parry

**Affiliations:** 1 Department of Pediatrics, Govt Medical College, Srinagar, Jammu and Kashmir 190010 India; 2 Department of Paediatrics, Govt Medical College Baramulla, Baramulla, Jammu and Kashmir India; 3 Department of Paediatrics, SKIMS Medical College Bemina, Srinagar, Jammu and Kashmir India

**Keywords:** Blood pressure, Children, Guidelines, Hypertension, Prevalence

## Abstract

Globally hypertension in adults is among the leading preventable cause of premature death, where a graded association from the childhood hypertension is well recognised. With the concurrent rise in obesity and pediatric hypertension (HTN) during the past decade in developed countries, a parallel trend is emerging in developing countries that has a potential for exponential rise in cardiovascular, cerebrovascular and renal tragedies. A cumulative incidence of pediatric HTN in China and India is 50–70 and 23% respectively, is quite disturbing. New guidelines for the detection, evaluation and management of hypertension in children and adolescents published in 2017, where a jump in prevalence of pediatric HTN is observed, rings a call to address this under-attended burning problem; for which a review in pediatric hypertension and its management is warranted.

## Introduction

Blood pressure (BP), is the pressure of the blood exerted on the arterial walls, produced by the contraction of the left ventricle against the resistance offered by arteries and arterioles that is required for the optimal body functioning, however, persistent high blood pressure (hypertension) is a global health issue. Globally, hypertension (HTN) is found to be major risk factor accounting for 10.2 million deaths and 208 million disability adjusted life years [1]. Evidence based exiting data published for both pediatrics and adults, has projected a graded association between increased blood pressure (BP) and risk of cardiovascular disease, end-stage renal disease, along with mortality [2–4].

Meta-analysis of more than 61 prospective studies from 1 million adults, showed that the risk of cardiovascular disease increased beginning at systolic BP levels less than 115 mmHg and diastolic BP levels less than 75 mmHg [5]. Considering 115/75 mmHg a normal BP for an adolescent corresponding to his/her age, height and sex; nevertheless, a consistent linear upward trend of this BP level forms the basis of adult HTN a leading cause of high cardiovascular, nervous system and kidney related morbidity and mortality.

### Prevalence

Globally the prevalence of hypertension is increasing and more than 1 billion people are hypertensive, and the increasing trends are witnessed more in low-income and middle-income countries [6, 7]. In our country (India), there is a steady increase in HTN prevalence from > 1% in 1960’s [8], 5–7% in 1990’s [9], and in 2013 it was 29.8% [10]. During last 10 years in USA, HTN has risen to 5% in adolescents; elevated BP (combination of prehypertension and HTN) increased up to 12.6% in girls and 19.2% in boys [11]; while using the fourth report on the diagnosis, evaluation, and treatment of high blood pressure in children and adolescents, a multicentre study in India showed the prevalence of 23% in systolic and/or diastolic hypertension among healthy school going 5–15 years old children [12]. In one of the landmark study in China, the overall prevalence of elevated blood pressure (≥95th percentile) among school age children (6–13 years) was 18.4%; 20.2% for boys and 16.3% for girls, with children aged 10–11 years having the highest prevalence [13]. In a cross- sectional study from Brazil involving 794 children, aged 6–13 years a prevalence of 7% of pediatric hypertension was reported [14], while in Japan, a prevalence 15.9% in 4th-grade boys and 15.8% in 4th-grade girls was observed [15].

### Definitions

According to the Fourth Report [16] the diagnosis of pediatric HTN was based on the distribution of BP values obtained from both normal and obese children, where as in new clinical practice guidelines (CPG) which is an update on 4th report, data was generated from healthy normal weight pediatric population and updated definitions [17], are detailed in Table 1, as under.

**Table 1:** Revised definition of pediatric blood pressure

BP/HTN	Age 1–< 13 Years	Age > 13 Years
Normal BP	<90th percentile	< 120/< 80 mmHg
Elevated BP	≥90th percentile to <95th percentile or 120/80 mmHg to <95th percentile (whichever is lower)	120/< 80–129/< 80 mmHg
Stage 1HTN	≥95th percentile to <95th percentile+ 12 mmHg or 130/80–139/89 mmHg (whichever is lower)	130/80–139/89 mmHg
Stage 2 HTN	≥95th percentile + 12 mmHg or ≥ 140/90 mmHg (whichever is lower)	≥140/90 mmHg

Adopted from Flynn JT, Kaelber DC, Baker-Smith CM, Blowy D, Carrole AE, Daniels SR et al. Clinical practice guideline for screening and management of high blood pressure in children and adolescents. Pediatrics 2017; 140: e20171904

### White coat hypertension (WCH)

BP ≥95th percentile in the office or clinical setting but <95th percentile outside of the office or clinical setting is considered as WCH which is more significant in pediatric population. It is confirmed by using Ambulatory Blood Pressure Monitoring (ABPM) where mean systolic blood pressure (SBP) and diastolic blood pressure (DBP) are <95th percentile, in addition to SBP and DBP load are < 25%; for age, sex, and height [18, 19].

### Masked hypertension (MH)

Again with aid of ABPM, it is the presence of hypertension despite normal office BP [20]. Obese and those with secondary forms of HTN especially chronic kidney disease (CKD) patients are at risk of MH [21].

### Primary hypertension (PH)

Also known as essential hypertension is defined as a blood pressure (BP) ≥95th percentile without an identifiable cause. Children with primary HTN are usually ≥6 years, overweight/obese, have positive family history and usually have systolic HTN [22].

### Secondary hypertension

It is the HTN with an identifiable cause, mostly seen in younger preschool children, where renal and urological disorders are the common causes and these children have usually diastolic HTN [22]. Common causes of pediatric HTN are shown in Table 2.

**Table 2:** Common causes of pediatric hypertension [23]

Age	Common Causes
Newborn	Renal artery thrombosis or embolus, Renal vein thrombosis, Congenital renal malformations, Coarctation of aorta, Renal artery stenosis, Bronchopulmonary dysplasia
Infancy to 6 years	Renal parenchymal disease, Renal artery stenosis, Coarctation of the aorta, Medications (corticosteroids, albuterol, pseudoephedrine), Endocrine causes
6–10 years	Renal parenchymal disease, Renal artery stenosis, Primary hypertension, Endocrine causes.
Adolescence	Primary hypertension, White coat hypertension, Renal parenchymal disease, Substance abuse (cocaine, amphetamines, methamphetamines, phencyclidine, methylphenidate, caffeine), Teen pregnancy, Endocrine causes.

### Methods of BP measurement

Accurate BP recording is must to address the problem at hand and parental concerns. It has been observed that anxiety and recent caffeine intake are causes of isolated BP elevation, reiterating the report from a study where only 56% of sample population had same stage HTN on 3 different occasions [24]. BP should be measured in the right arm by using standard measurement practices unless the child has atypical aortic arch anatomy, such as right aortic arch and coarctation of aorta or any similar anomaly. Various methods like auscultatory, aneroid, and oscillatory can be used for BP recording in children. However, for labelling a patient hypertensive is based on repeated auscultatory and ABPM measurement. It is prudent to use oscillatory method as a screening tool, where if BP is elevated, further evaluation is warranted.

### BP measuring devices

#### Auscultatory mercury sphygmomanometer

Normative values for blood pressure are based on auscultatory sphygmomanometry, which continues to be the preferred method for blood pressure estimation. After resting for 5-min, in relaxed environment, seated with back supported, feet on the floor, with no history of stimulant intake; a stethoscope is placed on brachial artery, proximal and medial to the antecubital fossa, below the bottom edge of the cuff, BP measurement is recorded. Age related proper sized cuff should be used for precise BP measurements in children. An estimated 80–100% length and 45–55% width of bladder to that of patients midarm circumference, while upper arm is held in neutral position with elbow flexed to 90° [25, 26] has been recommended.

Traditionally, blood pressure is assessed using the auscultatory technique (Korotkoff sounds) with the pressure in the cuff measured by mercury sphygmomanometer, which is recognised as, the ‘gold standard’. However, due to adverse environmental concerns like the maintenance and disposal problems, mercury as a component in BP measuring devices, has led to the imposition of bans in some European countries and its restricted use in health care, for which appropriate health and safety procedures should be followed including the availability of mercury spillage kits. Disposal of mercury should be performed in compliance with the appropriate environmental regulations in place.

### Oscillometric devices

Oscillometric devices are increasingly being used as they are user friendly, automatic with no digit preference. Although these devices measure the oscillations transmitted from disrupted arterial flow by using the cuff as a transducer to determine mean arterial pressure (MAP), and interpret the relation to proprietary algorithm for the MAP calculation [27]. These devices are very helpful in pre-schoolers and non-cooperative kids, and can well be used for screening purpose.

### Ambulatory blood pressure monitoring (ABPM)

ABPM devices measure BP outside the office setting and provides multiple readings over a period. It provides the mean, daytime, night-time ambulatory BP and detects the hidden variations of HTN. Based on existing data, ABPM is more accurate for the diagnosis of HTN than office/clinic-measured BP and is more predictive of future BP [28]. ABPM devices consist of BP cuff, a tubing that connects to a small wallet sized monitor, and circadian BP is recorded periodically (usually every 20–30 min) and then data is later downloaded to a computer for analysis, interpretation of which is done according to American Heart Association guidelines [29]. Here the patient is instructed to record wake-up time, sleep time and medication if any. Following classification has been suggested based on ABPM recordings in children [29, 30].

1.Normal BP: <90th percentile in office BP with <95th percentile mean ABPM and < 25% ambulatory BP load
WCH: Office BP with ≥95th percentile with <95th percentile mean ABPM and < 25% ambulatory BP load
MH: < 95th percentile in office with ≥95th percentile mean ABPM and ≥ 25% ambulatory BP load
Prehypertension: Office BP ≥90th percentile, with <95th percentile mean ABPM and ≥ 25% ambulatory BP load
Ambulatory HTN: Office BP ≥95th percentile, with ≥95th percentile mean ABPM and 25–50% ambulatory BP load
Severe Ambulatory HTN: Office BP ≥95th percentile, with ≥95th percentile mean ABPM and ≥ 50% ambulatory BP load

### Diagnosis of pediatric hypertension

#### History

As with other clinical entities, a thoughtful history and thorough clinical examination must be the first step for the evaluation of pediatric HTN. A detailed history starting from the conception, gestational events, foetal growth patterns, birth weight, maternal hypertension, [31] perinatal infection, and neonatal hospitalization must be noted followed by the information regarding nutritional history including early breast feeding [32], weaning, daily intake of amount of salt [12], fat, fast foods preferences, vegetable, fruit, and legumes. Like-wise history about the physical activity [33], screen time, and sleep-disordered breathing (SDB) should be sought [34]. Family history of hypertension [35], and renal diseases must be recorded. Similarly, psychosocial history regarding stressful childhood, early onset anxiety and depression [36], or obesity associated school bullying [37] must be noted.

#### Physical examination

To confirm various clues obtained from the history, physical examination starts with the assessment of general appearance like syndromic facies like (Willium’s, Cushing’s, Turner’s), along with weight, height, BMI, waist hip ratio, head circumference, and general growth. This is followed by vital sign measurements like pulse rate, radio femoral delay, difference in blood pressure in upper and lower limbs. Presence of pallor, proptosis, adenoids, thyromegaly, hirsutism, acne, café-au-lait spots, butterfly rash, lymphadenopathy, localised chest bulge, dyspnoea, murmur, apical heave, abdominal mass, palpable kidneys, ambiguous genitalia, hematuria, joint swellings, and muscle weakness must be looked for.

Screening of blood pressure should be carried if there is history of oligohydramnios, foetal USG documented renal/urological anomaly, prematurity, low birth weight, umbilical artery/vein catheterization, congenital heart disease, neurofibromatosis, tuberous sclerosis, ambiguous genitalia, recurrent urinary tract infections, features suggestive of renal disease, malignancy, organ transplant. Further action is warranted if the BP of the child or adolescent is above the values as depicted in Table 3.

**Table 3:** Blood Pressure requiring further evaluation [17]

Age (Years)	Boys		Girls	
	SBP (mmHg)	DBP (mmHg)	SBP (mmHg)	DBP (mmHg
1	98	52	98	54
2	100	55	101	58
3	101	58	102	60
4	102	60	103	62
5	103	63	104	64
6	105	66	105	67
7	106	68	106	68
8	107	69	107	69
9	107	70	108	71
10	108	72	109	72
11	110	74	111	74
12	113	75	114	75
≥13	120	80	120	80

Adopted from: Flynn JT, Kaelber DC, Baker-Smith CM, Blowy D, Carrole AE, Daniels SR et al. Clinical practice guideline for screening and management of high blood pressure in children and adolescents. Pediatrics 2017; 140: e20171904

However, applying this data which is extracted from the 4th report wherein overweight and obese children are excluded, will continue to serve as basic source, till quality indigenous data becomes available with us. While using the new Clinical Practice Guidelines 2017, the prevalence of confirmed hypertension among shorter children < 13 years old and taller 13+ years old children are more likely to be diagnosed with hypertension [38]. Concentrating around the impact of height on the pediatric HTN, it seems prudent to have indigenous reference normative data to draw the precision line between normotensive and hypertensive children among the Asian children.

### Laboratory evaluation

In general, basic work up like estimation of haemoglobin, renal functions, serum electrolytes, serum lipids, blood glucose, urinalysis routine and/or culture, spot protein to creatinine ratio and chest X-ray might be considered. In patients with BMI >95th percentile, HbA1c, ALT, AST, TSH, drug screen, and sleep study in SDB will be helpful [39]. In addition to above investigations echocardiography, [40, 41] renal ultrasonography, [42] CT/MR angiography are helpful for proper management of the pediatric hypertension [43, 44].

### Treatment

Goals for the pediatric hypertension must include prevention of target organ damage and occurrence of adult hypertension along with optimal BP maintenance among hypertensive children and adolescents. It has consistently been proven that Dietary Approach to Stop Hypertension (DASH) diet [45], and good physical activity of 40 min a day for 3 to 5 days a week [46], be initiated and continued before embarking on pharmacological treatment. The DASH diet includes multiple servings of fresh vegetables and fruits, whole grains, nuts and legumes; limiting foods high in sodium, sugars, and fats, with fair amount of lean protein products. Next step in regulating the HTN is pharmacologic treatment to those who fail lifestyle modifications, who have chronic kidney disease/ and/or diabetes mellitus with hypertension, symptomatic hypertension, and stage 2 hypertension. The current practice guidelines recommend that single antihypertensive drug with lowest dose must be initiated and upward titration or addition of second agent be sought after 2–4 weeks until BP reaches to <90th percentile or < 50th percentile in CKD patients. Hypertensive children with chronic kidney disease, proteinuria, or diabetes mellitus, an initial therapy of choice is angiotensin converting enzyme inhibitor or angiotensin receptor blockers, unless contraindicated. Otherwise, an angiotensin-converting enzyme inhibitor, angiotensin receptor blockers, long acting calcium channel blockers, and thiazide diuretics are appropriate initial agents.

### Follow up

The goal of the treating physician is to optimise the BP while the child is on pharmacotherapy and/or DASH approach. A 4–6 weekly follow up is recommended for the dose adjustments and drug modification in patients on pharmacotherapy. Once the BP is stabilized the frequency of visits can be extended to every 3 to 4 months. However, in DASH approach a 3–6 monthly follow up is required. It has been suggested that home BP measurement and repeat ABPM [47] is also required to assess current BP, compliance, drug adherence, and emergence of any complication.

## Conclusion

Rising prevalence of pediatric HTN carrying global health dimensions, needs early identification and appropriate management, a goal-oriented step wise progress with heightened awareness of this entity shall be envisioned, where primary prevention could stand tall before all measures.

## Data Availability

Published medical data mostly of last 5 years.
